# Osteoblast-Derived Vesicle Protein Content Is Temporally Regulated During Osteogenesis: Implications for Regenerative Therapies

**DOI:** 10.3389/fbioe.2019.00092

**Published:** 2019-05-01

**Authors:** Owen G. Davies, Sophie C. Cox, Ioannis Azoidis, Adam J. A. McGuinness, Megan Cooke, Liam M. Heaney, Edward T. Davis, Simon W. Jones, Liam M. Grover

**Affiliations:** ^1^School of Sport, Exercise and Health Sciences, Loughborough University, Loughborough, United Kingdom; ^2^School of Chemical Engineering, University of Birmingham, Birmingham, United Kingdom; ^3^Physical Sciences for Health Doctoral Training Centre, University of Birmingham, Birmingham, United Kingdom; ^4^Royal Orthopaedic Hospital, Birmingham, United Kingdom; ^5^Institute of Inflammation and Ageing, University of Birmingham, Birmingham, United Kingdom

**Keywords:** vesicle, mineralization, annexin, collagen, osteoblast, nano

## Abstract

Osteoblast-derived extracellular vesicles (EV) are a collection of secreted (sEVs) and matrix-bound nanoparticles that function as foci for mineral nucleation and accumulation. Due to the fact sEVs can be isolated directly from the culture medium of mineralizing osteoblasts, there is growing interest their application regenerative medicine. However, at present therapeutic advancements are hindered by a lack of understanding of their precise temporal contribution to matrix mineralization. This study advances current knowledge by temporally aligning sEV profile and protein content with mineralization status. sEVs were isolated from mineralizing primary osteoblasts over a period of 1, 2, and 3 weeks. Bimodal particle distributions were observed (weeks 1 and 3: 44 and 164 nm; week 2: 59 and 220 nm), indicating a heterogeneous population with dimensions characteristic of exosome- (44 and 59 nm) and microvesicle-like (164 and 220 nm) particles. Proteomic characterization by liquid chromatography tandem-mass spectrometry (LC-MS/MS) revealed a declining correlation in EV-localized proteins as mineralization advanced, with Pearson correlation-coefficients of 0.79 (week 1 vs. 2), 0.6 (2 vs. 3) and 0.46 (1 vs. 3), respectively. Principal component analysis (PCA) further highlighted a time-dependent divergence in protein content as mineralization advanced. The most significant variations were observed at week 3, with a significant (*p* < 0.05) decline in particle concentration, visual evidence of EV rupture and enhanced mineralization. A total of 116 vesicle-localized proteins were significantly upregulated at week 3 (56% non-specifically, 19% relative to week 1, 25% relative to week 2). Gene ontology enrichment analysis of these proteins highlighted overrepresentation of genes associated with matrix organization. Of note, increased presence of phospholipid-binding and calcium channeling annexin proteins (A2, A5, and A6) indicative of progressive variations in the nucleational capacity of vesicles, as well as interaction with the surrounding ECM. We demonstrate sEV-mediated mineralization is dynamic process with variations in vesicle morphology and protein content having a potential influence on developmental changes matrix organization. These findings have implications for the selection and application of EVs for regenerative applications.

## Introduction

Bone formation is a carefully orchestrated and well-regulated process whereby inorganic mineral is deposited on a collagenous matrix. Early mineral deposition is principally coordinated by osteoblasts, which modulate local phosphate ion concentrations and drive osteogenesis. Despite significant advancement in musculoskeletal biology, current understanding of the processes by which mineral is first deposited and propagated within the extracellular matrix (ECM) remain incompletely understood (Boskey, 1998). Early studies postulated that matrix mineralization was largely a passive and cell-independent process whereby non-collagenous proteins confined to gap zones function as sites for the localization of ions that drive mineral nucleation (Bonucci, 2012). However, two highly significant ultrastructural studies published in 1967 identified the active contribution of nano-sized extracellular vesicles (EVs, 50–400 nm), which functioned as loci for early mineral nucleation and propagation in growth plate cartilage (Anderson, 1967; Bonucci, 1967). It was subsequently demonstrated that mineral crystallized in and around EVs, forming the spheroidal nodules characteristic of mineralization both *in vitro* and *in vivo*. It has since been shown that these particles are released into the developing ECM where they determine the specific and localized pattern of nodular mineralization observed in developing bones (Anderson, 1995). Mature nodules then fuse to generate seams of woven bone (Marvaso and Bernard, 1977). Recent evidence suggests that this process is dynamic, progressing from an initial amorphous phase through a series of precursors and that this transition is, in part, facilitated by EVs (Mahamid et al., 2010).

Since these initial studies, EVs have been identified in a wide range of tissues and biofluids. This has resulted in the establishment of the International Society for Extracellular Vesicles (ISEV) and the subsequent publication of position statements outlining criteria for defining these complex bioactive nanoparticles (Théry et al., 2019). However, advancement in vesicle biology and nomenclature has largely occurred in parallel to developments in bone matrix biology. Vesicles identified within the extracellular matrix of bone are routinely defined matrix vesicles (MV) with a size distribution ranging from 50–400 nm. Aligning this definition with current vesicle nomenclature, it would be reasonable to hypothesize that these vesicles comprise a heterogeneous mixture of exosomes (30–150 nm) and microvesicles (100–500 nm) that may have divergent roles in directing mineralization and intercellular communication in developing bone (Shapiro et al., 2015). Furthermore, it is now understood that in addition to matrix-associated vesicles, osteoblasts cultured *in vitro* also generate a population of secreted vesicles that can be isolated the surrounding culture medium (Nair et al., 2014; Davies et al., 2017). We have previously demonstrated that these secreted EVs (sEVs) are enriched in annexin calcium-binding proteins and that the exogenous delivery of these bioactive particles can significantly enhance osteogenesis in mesenchymal stem cell (MSC) cultures (Davies et al., 2017). However, if these pro-osteogenic sEVs are to be applied therapeutically additional work is required to define how their properties vary over the course of mineralization and better understand how their properties align with the more comprehensively characterized MVs (Shapiro et al., 2015).

Vesicles derived from different tissue sources are uniquely adapted to fulfill the requirements of that tissue. As such, vesicles will contain specific protein, lipid and nucleic acid signatures indicative of their tissue of origin. Within developing bone, osteoblast-derived vesicles have been shown to function in early mineralization events preceding endochondral ossification. To fulfill their function, these particles are enriched in membrane lipids (e.g., cholesterol, sphingomyelin and phosphatidylserine), calcium binding proteins (e.g., annexins-A1, -A2,−5, and –A6) and phosphate converting enzymes (e.g., tissue non-specific alkaline phosphatase, TNAP, and nucleotide pyrophosphatase phosphodiesterase, Enpp) (Kirsch et al., 2000; Davies et al., 2017). Together this specialized collection of lipids and proteins comprises the nucleational-core-complex (NCC), which facilitates the uptake, binding and organization of calcium and phosphate ions to initiate mineral formation, expansion and crystallization (Wu et al., 1993). It is proposed that EVs bud from the osteoblast plasma membrane and accumulate calcium and phosphate ions extracellularly, providing an optimized niche for the transition to crystalline apatite that eventually ruptures the vesicle and is deposited on the collagenous ECM (Davies et al., 2017; Hasegawa et al., 2017). Additionally, internal sources of polyphosphate, such as ATP, are also considered to play a significant role in coordinating osteogenesis both as a source of inorganic phosphate and through their action on purinergic signaling pathways (Orriss et al., 2013). However, much of the data surrounding existing theory has been collected in studies of matrix-bound vesicles, where MVs have been digested from the collagenous matrix (Bottini et al., 2018). Consequently, limited information exists regarding the precise temporal contribution of sEVs to matrix mineralization. Since the production and isolation of sEVs is less laborious and time-consuming than MVs they present an appealing source of particles for regenerative applications and investment is required to comprehensively characterize their temporal roles in matrix mineralization.

To date, we and others have documented the capacity of vesicles isolated from the media of differentiating osteoblasts to function as sites of nucleation within mineralizing tissues and have begun to define a mechanism of action based on their biological content (Davies et al., 2017). However, if we are to fully understand their contribution to hard tissue matrix development and potentially exploit these bioactive particles to enhance bone regeneration it is essential that we begin to understand how their function changes over the course of mineralization. In this study we morphologically and proteomically characterize vesicles secreted by primary human osteoblasts under defined culture conditions during mineralization in order to define their temporal effects during osteogenesis.

## Materials and Methods

### Primary Osteoblast Isolation and Culture

This study was approved by the University of Birmingham UK Research Ethics Committee (Reference: 16/SS/0172). Written informed consent was obtained from all participants. Human osteoblast cells were obtained from donated human bone following joint replacement surgeries and all experiments were carried out in triplicate using cells derived from three independent donors. Cartilage was removed from the femoral condyles and tibial plateaux. The exposed trabecular bone was cut into small chips of ~2 mm^3^. To remove contaminating adipose, chips were washed 3 times in 30 mL mineralization media (MM) comprising of minimal essential media α modification (α-MEM) (Sigma-Aldrich, UK) supplemented with 10% fetal bovine serum (FBS), 100 units/mL penicillin (Sigma-Aldrich, UK), 100 μg/mL streptomycin (Sigma-Aldrich, UK), 2 mM L-glutamine (Sigma-Aldrich, UK), 1% non-essential amino acids (Invitrogen, UK), 2 mM β-glycerophosphate (Sigma-Aldrich, UK), 50 μg/mL ascorbic acid (Sigma-Aldrich, UK), 10 nM dexamethasone (Sigma-Aldrich, UK). Chips were then placed into a T125 culture flask (Nunc, UK) with fresh MM and cultured, replacing media every 3–4 days, to encourage outgrowth of osteoblasts. Bone chips were removed after 10–14 days and remaining osteoblasts were cultured, typically reaching confluence after 21 days, at which point the cells were passaged. Non-mineralizing controls were cultured in growth medium (GM) that comprised of minimal essential media α modification (α-MEM) (Sigma-Aldrich, UK) supplemented with 10% fetal bovine serum (FBS), 100 units/mL penicillin (Sigma-Aldrich, UK), 100 μg/mL streptomycin (Sigma-Aldrich, UK), 2 mM L-glutamine (Sigma-Aldrich, UK) and 1% non-essential amino acids (Invitrogen, UK).

### qRT-PCR Quantification of Gene Expression

Comparative expression of osteogenic marker genes was evaluated in osteoblast isolated from three independent donors to asses variability between donors. Bone chips were washed in sterile PBS before being snap frozen in liquid nitrogen. They were then powdered using a cryogenic grinder (6775 Freezer Mill, SPEX SamplePrep, Stanmore, UK) before RNA isolation using TRIzol reagent (ThermoFisher, UK). Briefly, 1 mL TRIzol reagent was added to 100 mg of powdered tissue and homogenized using a TissueRuptor (Qiagen, UK). Chloroform was added to separate the RNA from the DNA and protein phases. The RNA was then precipitated using isopropanol overnight at −20°C. The precipitated RNA was centrifuged, and the pellet washed with 70% ethanol before drying and then resuspending in RNAse-free water. The concentration and quality of RNA was verified using the Nanodrop 2000 (ThermoFisher, UK). 260/280 ratios of >1.8 were deemed suitable for PCR analysis.

For qRT-PCR, OneStepPLUS SYBR Green master mix, custom primers (sequences provided in Table 1) and the housekeeping gene (B-actin) were designed and made by PrimerDesign (Southampton, UK). PCR was run on a BioRadCFX384 for 50 amplification cycles before the relative expression of each gene was calculated using the ddCT method.

**Table 1:** Primer sequences and accompanying gene accession numbers.

**Gene**	**Forward**	**Reverse**	**Accession No**.
ALP	CTTGGGCAGGCAGAGAGTA	AGTGGGAGGGTCAGGAGAT	NM_000478
BGLAP	GGCACCCTTCTTTCCTCTTC	TTCTGGAGTTTATTTGGGAGCA	NM_199173
BSP	GAGGTGATAGTGTGGTTTATGGA	TGATGTCCTCGTCTGTAGCA	NM_00104005
COL1A1	AGACAGTGATTGAATACAAAACCA	GGAGTTTACAGGAAGCAGACA	NM_000088

### Isolation of Secreted Extracellular Vesicles

Primary human osteoblasts isolated from three independent donors were cultured at passage 2 for a total period of 21 days. Cell culture medium incorporated FBS that has been ultracentrifuged at 120,000 × g for a period of 18 h to deplete any naturally occurring vesicles that may compromise the quality of downstream analyses (Shelke et al., 2014). sEVs were isolated from primary human every 2–3 days and pooled into three respective groups: week 1, 2, and 3. Cells and cellular debris was removed by centrifuging each sample at 2000 × g for 30 min. Five millilitre of the supernatant was carefully transferred to a sterile polycarbonate ultracentrifuge tubes (Beckman Coulter, UK) containing 2.5 mL commercial total exosome isolation reagent (ThermoFisher, UK). The solution was mixed thoroughly and incubated at 4°C to allow for sEV precipitation. Following incubation, the samples were transferred to an Avanti J-26S XPI high performance centrifuge (Beckman Coulter, UK) and spun at 10,000 × g for 60 min at 4°C in line with the manufacturer's guidelines. The supernatant was discarded, and each pellet resuspended in 300 μL filtered PBS. Fifty microliter were stored at −80°C before analysis.

### sEV Sizing and Quantitation

Total protein concentration was determined using the Pierce BCA protein assay kit (ThermoFisher, UK) and confirmed by Nanodrop spectrophotometry (ThermoFisher, UK). Particle concentration was determined using a Nanosight LM10 (Malvern Panalytical, UK) and size distribution analyzed by Dynamic Light Scattering using the Zetasizer HPPS (Malvern Panalytical, UK). All experiments were carried out in triplicate.

### Transmission Electron Microscopy

A suspension of sEVs was made in sterile filtered PBS. A single drop of the suspension (~20 μL) was deposited onto a carbon film grid (Agar Scientific, UK) and left to air dry for a period of 1 min. To remove excess PBS the grid was gently blotted using tissue paper through capillary action. Negative staining was achieved by placing a single drop (~20 μL) of uranyl acetate solution onto the dry grid. Samples were imaged using a JEM 3200FX transmission electron microscope (Joel, USA) using a voltage of 80 kV.

### Alkaline Phosphatase Assay

Cellular ALP levels were quantified using the SensoLyte^®^ pNPP Alkaline Phosphatase Assay Kit (Anaspec, UK) according to the manufacturer's instructions. Briefly, cell monolayers were washed twice using 1x assay buffer provided. Cells were detached from the surface of the culture plate in the presence of 200 μL permeabilization buffer using a cell scraper. The resulting cell suspension was collected in a microcentrifuge tube, incubated at 4°C for 10 min under agitation, and then centrifuged at 2500 × g for 10 min. Supernatant was transferred to a 96 well plate where it was combined with an equal volume of pNPP substrate. Absorbance was measured at 405 nm using a plate reader. ALP levels were normalized to cell number, which was determined using Trypan blue cell counting.

### Alizarin Red Staining and Quantification

Human osteoblast cultures were incubated for a period of 21 days in MM, with medium changes performed every 2–3 days. Calcium-rich deposits were visualized using 40 mM alizarin red (AR - Sigma-Aldrich, UK) stain, which was dissolved in acetic acid and adjusted to pH 4.2 using 5M ammonium hydroxide. Bound AR was quantified through acetic acid extraction and colorimetric detection at 405 nm. AR concentration were normalized to cell number, which was determined using Trypan blue cell counting.

### Mass Spectrometry

Mass spectrometry analysis was performed on sEVs isolated from three separate donors. Each was analyzed in triplicate to limit technical variation. sEV proteins were digested by FASP with LysC and trypsin as described previously (Wisniewski et al., 2009). The peptides generated were then separated by nano-flow reversed-phase liquid chromatography coupled to Q Exactive Hybrid Quadrupole-Orbitrap mass spectrometer (Thermo Scientific, UK). Peptides were loaded on a C18 PepMap100 pre-column (300 μm I.D. × 5 mm, 3 μm C18 beads; Thermo Scientific) and separated on a 50 cm reversed-phase C18 column (75 μm I. D., 2 μm C18 beads). Separation was conducted with a linear gradient of 7–30% B 120 min at a flow rate of 200 nL/min (A: 0.1% formic acid, B: 0.1% formic acid in acetonitrile). All data was acquired in a data-dependent mode, automatically switching from MS to collision-induced dissociation MS/MS on the top 10 most abundant ions with a precursor scan range of 350–1650 m/z. MS spectra were acquired at a resolution of 70,000 and MS/MS scans at 17,000. Dynamic exclusion was enabled with an exclusion duration of 40 s. The raw data files generated were processed using the MaxQuant (version 1.5.0.35) software, integrated with the Andromeda search engine as described previously (Cox and Mann, 2008; Cox et al., 2011).

Resulting MS/MS spectra were searched against the human proteome (UniProt 2013/04/03), precursor mass tolerance was set to 20 ppm, variable modifications defined as acetylation (protein N-terminus) and oxidation (M), carbamidomethylation (C) was defined as fixed modification. Enzyme specificity was set to trypsin with a maximum of 2 missed cleavages. Protein and peptide spectral matches (PSM) false discovery rate (FDR) was set at 0.01 and match between runs was applied. Finally, only proteins identified in 2 biological replicates with a minimum of 2 spectral counts (SC) in one biological replicate were included for analysis. For proteins to be considered specific to a particular fraction they must have exhibited combined SC ≥ 5.

Differential protein abundance analysis was performed with Perseus (version 1.5.5.3). In brief, Log_2_-transformed protein intensity distributions were median-normalized and a *t*-test was used to assess the statistical significance of protein abundance fold-changes. *P*-values were adjusted for multiple hypothesis testing with the Benjamini-Hochberg correction (Hochberg and Benjamini, 1990).

### Multivariate Statistics to Highlight Candidate Proteins

Multivariate analyses were performed using SIMCA (v14.1, MKS Umetrics AB, Umeå, Sweden). Protein abundances were Log2-transformed, median-normalized and an arbitrary minimum value assigned to instances where the measured protein intensity was zero. All data were Pareto scaled prior to analyses. Initially, an unsupervised principal components analysis (PCA) was performed on all measured proteins and a 3-dimensional score plot produced to visualize the profile of each experimental arm (weeks 1, 2, and 3). In addition, supervised analyses were performed using orthogonal partial least squares-discriminate analyses (OPLS-DA) to investigate between-group differences in protein profiles for weeks 1 vs. 2, 1 vs. 3 and 2 vs. 3. Peptides considered as major contributors to the separation of sample groupings were identified by assessing the relevant S-plot.

### Bioinformatic Analysis

The protein annotation through evolutionary relationship (PANTHER, http://www.pantherdb.org/) classification system (version 9.0) was used for gene ontology (GO) annotation of biological pathways, molecular mechanisms and cellular compartments of proteins.

### Data Analysis and Statistics

*T*-Tests and ANOVA statistical analyses were performed using MiniTAB® v.16 (MiniTAB Inc., State College, PA, USA). *p* < 0.05 were defined as statistically significance.

## Results

**Figure 1:**
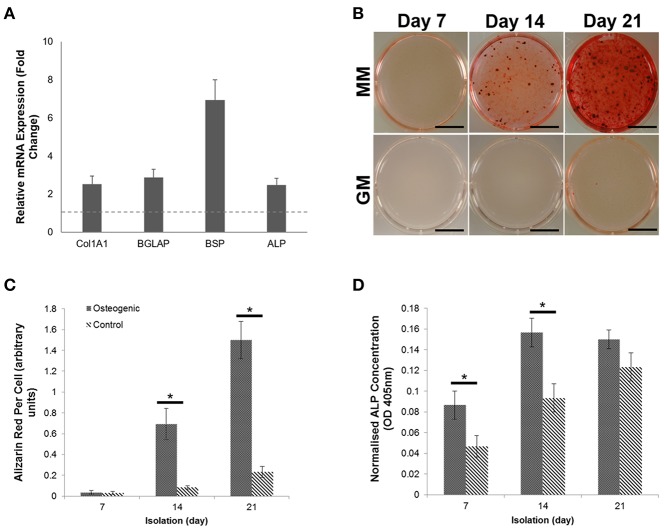
Characterization and differentiation of human primary osteoblasts derived from three separate donors toward a mineralizing phenotype over a period of 21 days. **(A)** Gene expression profiling of primary human osteoblasts showed upregulation of four osteogenic markers. **(B)** Differentiation of osteoblasts cultures in the presence of growth medium (GM) and mineralization medium (MM) containing 10 nM β-glycerophosphate and 50 μg/mL L-ascorbic acid over a period of 7, 14, and 21 days induced extracellular matrix mineralization, which was visualized using Alizarin red calcium stain. **(C)** Visual differences in mineralization observed using Alizarin red were confirmed semi-quantitatively. **(D)** The concentration of Alkaline phosphatase (ALP) was calculated per cell. Significant increases in ALP concentration were observed at days 7 and 14. Scale bars = 15 mm, *p ≤ 0.05. *N* = 3.

Primary human osteoblasts were isolated from trabecular bone obtained during elective hip arthroplasty surgeries (UK National Research Ethics Committee Reference: 16/SS/0172). Isolated osteoblasts were characterized prior to expansion for the expression of genes indicative of phenotype. All four genes (Col1A1, BGLAP, ALP, and BSP) were shown to be upregulated when compared with a baseline housekeeping gene, GAPDH (Figure 1A). The presence of calcium deposition within the developing matrix was observed using alizarin red (AR) staining (Figure 1B). Visualization and quantification of AR staining revealed that mineral deposition was limited in day 7 cultures but showed statistical significance (*p* < 0.05) when compared with non-osteogenic controls at days 14 (8.32-fold increase) and 21 (6.43-fold increase) (Figure 1C). The expression of the pyrophosphate hydrolysing enzyme alkaline phosphatase (ALP) was significantly increased (*p* < 0.05) at day 7 and 14 when compared with the control (Figure 1D), further confirming induction of differentiation and mineralization in primary osteoblast cultures.

**Figure 2:**
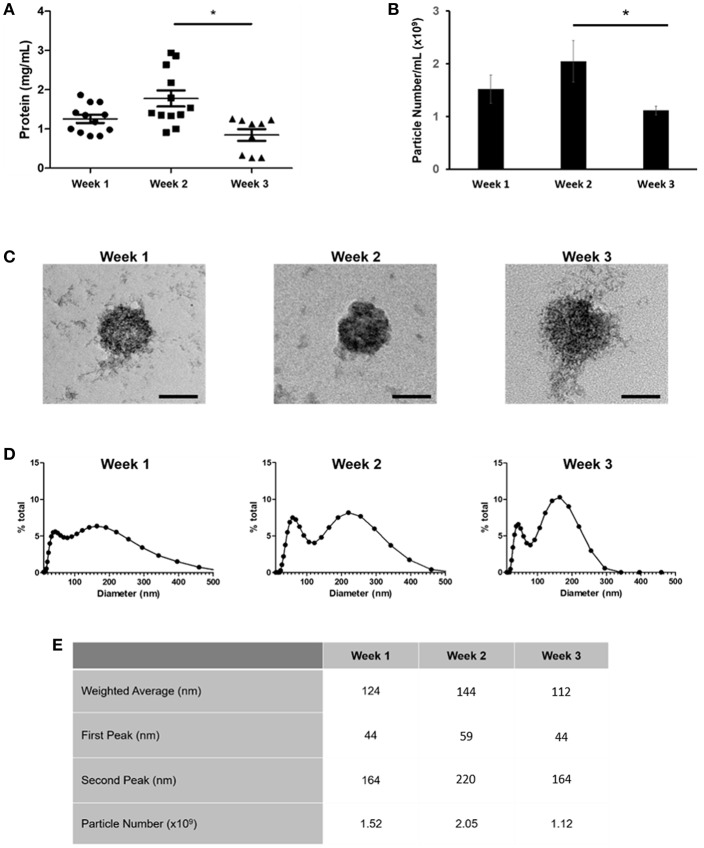
Comparison of sEVs released by mineralising primary osteoblasts derived from three separate donors into culture medium over a period of 21 days. Three repeats were performed for each donor. EVs were isolated during the first, second, and third week of differentiation and **(A)** total protein and **(B)** particle number quantified. **(C)** sEVs were visualized using TEM. Changes in sEV morphology were apparent, with visual indications of rupture evident during week 3. **(D)** Relative sEV diameter was assessed using dynamic light scattering (DLS) **(E)** Median peak intensities and weighted averages for sEVs isolated at each time point. Scale bars = 50 nm, *p ≤ 0.05. *N* = 3.

We have previously shown that sEVs function as sites of mineral nucleation in developing matrix. However, there is currently little understanding of how the contribution of these particles changes as cells progress from an immature to a mature state. To begin to answer this question we isolated sEVs from the culture medium of mineralizing primary human osteoblasts derived from three independent donors over the course of 1, 2, and 3 weeks. Temporal differences in total protein content were observed with a significant (*p* < 0.05) decline in protein concentration detected during the final week of mineralization (Figure 2A). Nanoparticle tracking analysis (NTA) was applied to determine how total protein content related to total particle number (Figure 2B). In alignment with protein concentration, the number of particles isolated was found to decrease in mature cultures, with a significant (*p* < 0.05) decline in particle number observed between weeks 2 (2.05 × 10^9^) and 3 (1.12 × 10^9^). These findings may suggest that most of the protein was associated with vesicles with limited indication of the presence of non-vesicular proteins. The presence of sEVs at each time point was visually confirmed using transmission electron microscopy (TEM) (Figure 2C and Supplementary Image 1). Differences in vesicle morphology were noted, with vesicles isolated during week 3 displaying a less organized structure and visual evidence of rupture which is perhaps indicative of a decline in membrane integrity and the release of luminal content (Figure 2C). Particles from all three isolations featured an electron dense core, which appeared to become more organized at week 2. Particle size measurement was achieved using dynamic light scattering (DLS) and indicated a bimodal size distribution over the course of osteoblast differentiation (Figures 2D). Particle polydispersity decreased over time and bimodality become increasingly evident with peaks becoming increasingly distinct as mineralization advanced. At week 3 (330 nm) maximum particle size decreased relative to weeks 1 and 2 (>500 nm). Weighted averages calculated for particles showed no statistical change over the course of 3 weeks, with mean sizes of 124, 144, and 112 nm, respectively (Figure 2E).

**Figure 3:**
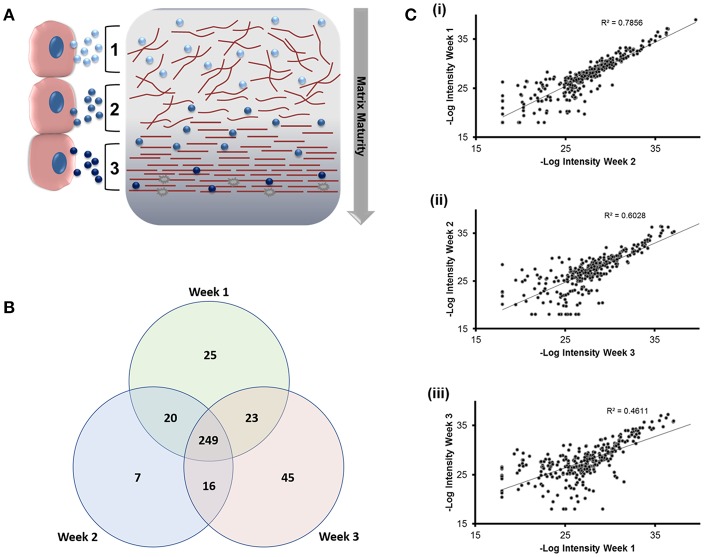
Analysis of differentially expressed proteins localized to osteoblast sEVs identified using LC-MS/MS. sEVs were isolated from three separate donors with three repeats performed for each donor. **(A)** Schematic representation of the relationship between sEVs in relation to the maturity and organization of the mineralized matrix. **(B)** Venn diagram depicting specific and non-specific proteins localized to sEVs isolated during 1, 2- and 3-weeks mineralization. **(C)** MS peak intensity plots comparing -Log_10_ MS-peak intensities of proteins associated with sEVs during weeks 1 (i), 2 (ii), and 3 (iii) of differentiation and their associated Pearson's correlation values. *N* = 3.

We next applied nano-liquid chromatography-tandem mass spectrometry (LC-MS/MS) to determine how the total number of proteins associated with osteoblast sEVs changed as mineralization advanced and the extracellular matrix became demonstrably more calcified (Figure 3A). Our data revealed the presence of 310 (week 1), 292 (week 2), and 281 (week 3) proteins, respectively (Figure 3B). A total of 249 proteins were shared throughout the 21-day culture period. 77 proteins were found to be exclusively localized to sEVs isolated at different time points during osteoblast differentiation, with 25 identified at week 1, 7 at week 2, and 45 at week 3. Analysis of relative protein intensity revealed a 79% correlation in MS peak measurements between 269 common proteins at weeks 1 and 2 (Figure 3Ci). This trend declined as mineralization progressed, with 60% correlation in the intensity of proteins co-expressed at weeks 2 and 3 (Figure 3Cii), while only a 46% correlation was observed between weeks 1 and 3 (Figure 3Ciii).

**Figure 4:**
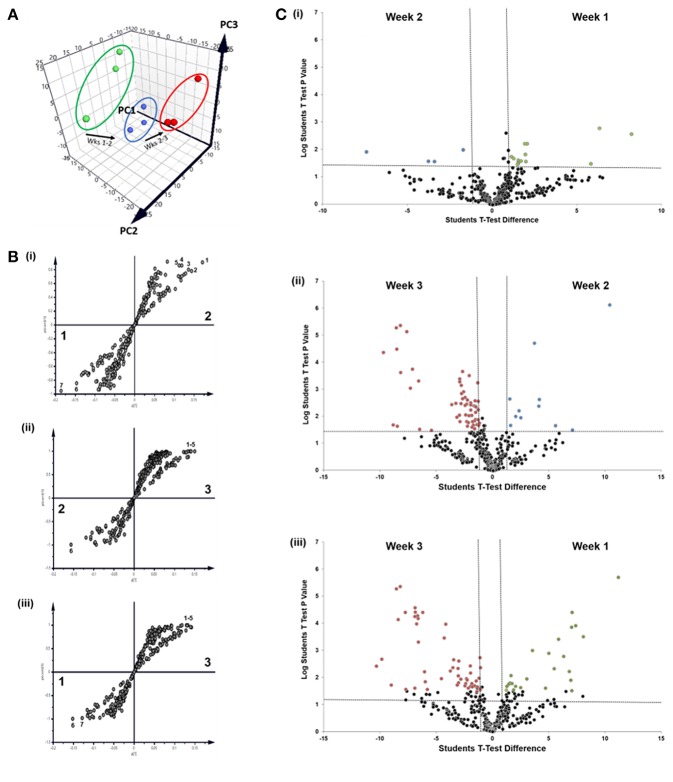
Comparative analysis of proteins localized to osteoblast sEVs during matrix mineralization. **(A)** Principal component analysis of total protein intensity. **(B)** Multiple orthogonal partial least squares-discriminant (OPLS-DA) analysis of between-group differences in MS protein intensity to identify differentially expressed proteins between sEVs isolated from the culture medium of mineralising osteoblasts at weeks 1, 2, and 3. Discriminatory proteins identified between (**B**i) weeks 1 and 2 included 1, plectin; 2, galectin; CD59 glycoprotein; 4, aminopeptidase N; 5, chitinase-3-like protein 1; 6, collagen alpha-1 XVI chain; CD81 antigen; (**B**ii) weeks 2 and 3: 1, glia-derived nexin; 2, annexin A6; 3, ApoE; 4, protein transport protein Sec23A; 5, AP-2 complex subunit alpha-1/2; and (**B**iii) weeks 1 and 3: 1, plectin; 2, translational endoplasmic reticulum ATPase; 3, Annexin A6; 4, inactive serine protease PAMR1; 5, protein transport protein Sec23A; 6, reticulocalbin-3; 7, endoplasmin. **(C)** Volcano plots displaying Log_2_ values for protein fold-change against Log_10_ false discovery rate (FDR). Only proteins with a Log_2_ fold change of >1 and a *p* < 0.05 were considered to be statistically significant.

We next sought to identify proteins that were most differentially regulated in sEVs throughout the course of mineralization and identify whether these changes were distinct between weeks 1, 2, and 3. To visualize global proteomic changes for secreted osteoblast EVs across the 3-week period, a principal component analysis (PCA) of total protein intensity was applied. PCA highlighted a clear spatial distinction between MS protein spectra collected at each time point (Figure 4A). To further understand the proteins that demonstrated the greatest discriminatory power between time points, multiple orthogonal partial least squares-discriminant (OPLS-DA) analyses were performed (i.e., weeks 1v2, 1v3, 2v3). Separation of time points was seen in all 2-dimensional score plots corresponding to OPLS-DA analyses (data not shown) and S-plots were consulted for the up- and down-regulated proteins that had the greatest influence on the multivariate model (Figure 4B). Volcano plots were generated to identify vesicle-associated proteins that were significantly upregulated at early, intermediate and mature phases of osteoblast differentiation (Figure 4C). Only proteins with a Log_2_ fold change of >1 and a *p* < 0.05 were considered to be statistically significant. Analysis revealed that relatively few significant differences could be observed between the relative protein profiles of vesicles isolated at week 1 and 2 (Figure 4Ci). 17 proteins were found to be significantly upregulated between these two isolations, with 4 upregulated at week 2 (blue: S100A6, PLEC, ANPEP, CHI3L1) and 13 upregulated at week 1 (green: CD81, FBN2, NID2, RELN, NUTF2, THBS1, LGALSL, MFGE8, COL4A1, COL5A2, COL16A1, PCOLCE2, ADK, MYH9, FBN1, HSPG2). As primary osteoblasts differentiated and generated a mineralized matrix the number of differentially expressed proteins increased significantly, with 75 and 73 proteins significantly upregulated between vesicles isolated at weeks 2 and 3 (12 at week 2, 63 at week 3), and weeks 1 and 3 (26 at week 1, 47 at week 3), respectively (Figure 4Cii,iii). When including proteins exclusive to week 3 vesicles, a total of 116 were found to be differentially upregulated. Of these, 65 proteins were non-exclusively up-regulated, 22 were up-regulated relative to week 1 and 29 relative to week 2. Comprehensive details of these proteins can be found in Table 2.

**Table 2:** Proteins identified as exclusive to or significantly upregulated in sEVs isolated from the culture medium of human osteoblasts derived from three separate donors during the third week of mineralization.

**Gene name**	**UniProt ID**	**Protein name**	**Week 1^a^**	**Week 2^a^**	**Week 3^a^**	**Week 1^b^**	**Week 2^b^**	**Week 3^b^**
**UPREGULATED IN WEEK 3**								
NT5E	P21589	5-nucleotidase	20.3	25.2	27.9	0	2	5
YWHAE	P62258	14-3-3 protein epsilon	19.6	22.0	25.4	1	1	1
MMP2	P08253	72 kDa type IV collagenase	30.3	31.2	32.7	15	16	23
ANPEP	P15144	Aminopeptidase N	24.8	28.6	30.9	3	12	14
ANXA2	P07355	Annexin A2	29.5	30.4	32.3	12	15	17
ANXA6	P08133	Annexin A6	18.0	18.0	26.5	0	0	3
APOC3	P02656	Apolipoprotein C-III	20.7	23.3	30.0	0	1	1
APOE	P02649	Apolipoprotein E	22.4	18.0	26.5	4	3	7
BSG	P35613	Basigin	18.0	18.0	24.2	0	0	1
CAV1	Q03135	Caveolin-1	18.0	18.0	25.0	0	0	1
CPM	P14384	Carboxypeptidase M	18.0	18.0	24.7	0	0	1
CTSD	P07339	Cathepsin D	18.0	22.3	24.9	0	0	1
CD44	P16070	CD44 antigen	25.4	26.0	27.2	74	45	87
CD59	P13987	CD59 glycoprotein	25.2	20.0	26.8	1	0	1
CHI3L1	P36222	Chitinase-3-like protein 1	25.4	28.8	30.0	2	8	9
COPB2	P35606	Coatomer subunit beta	18.0	18.0	24.6	0	0	2
COL6A1	P12109	Collagen alpha-1(VI) chain	33.8	34.4	35.6	29	32	33
COL6A3	P12111	Collagen alpha-3(VI) chain	35.7	35.1	37.0	119	108	131
C1QTNF1	Q9BXJ1	Complement C1q tumor necrosis factor-related protein 1	18.0	18.0	24.6	0	0	1
CXCL6	P80162	C-X-C motif chemokine 6	26.6	27.9	30.3	3	5	5
DCN	P07585	Decorin	33.6	33.4	34.5	14	14	17
EEF1G	P26641	Elongation factor 1-gamma	22.4	20.1	25.4	1	0	1
EIF4A1	P60842	Eukaryotic initiation factor 4A-I	26.8	25.8	26.2	1	0	2
FSCN1	Q16658	Fascin	20.4	19.6	24.3	0	0	1
FTH1	P02794	Ferritin heavy chain	26.1	26.4	28.0	1	2	3
FIBIN	Q8TAL6	Fin bud initiation factor homolog	20.4	18.0	24.2	0	0	1
LGALS1	P09382	Galectin-1	20.4	26.5	29.3	0	3	4
GSN	P06396-2	Gelsolin	22.1	20.2	24.3	1	1	1
SERPINE2	P07093-2	Glia-derived nexin	31.2	31.2	33.1	0	0	1
GDF6	Q6KF10	Growth/differentiation factor 6	21.2	18.0	25.1	1	0	1
GNB2	P62879	Guanine nucleotide-binding protein G(I)/G(S)/G(T) subunit beta-2	18.0	22.6	24.1	0	1	2
ISLR	O14498	Immunoglobulin superfamily containing leucine-rich repeat protein	20.5	21.8	24.6	0	1	1
KPNB1	Q14974	Importin subunit beta-1	22.6	18.0	26.2	1	0	1
PAMR1	Q6UXH9	Inactive serine protease PAMR1	18.0	19.9	26.4	0	0	2
IGFBP2	P18065	Insulin-like growth factor-binding protein 2	26.1	26.9	28.4	9	9	10
ITGAV	P06756	Integrin alpha-V	19.6	18.0	24.4	0	0	1
KATNAL2	Q8IYT4	Katanin p60 ATPase-containing subunit A-like 2	18.0	19.9	24.9	0	0	1
LAMA1	P25391	Laminin subunit alpha-1	25.4	26.1	27.3	2	2	4
LAMA4	Q16363	Laminin subunit alpha-4	31.0	31.3	32.8	26	28	40
LAMB1	P07942	Laminin subunit beta-1	31.7	32.6	34.0	40	46	58
LUM	P51884	Lumican	19.9	22.9	25.8	0	1	1
MAMDC2	Q7Z304	MAM domain-containing protein 2	20.4	20.3	26.7	0	0	4
MFAP2	P55001	Microfibrillar-associated protein 2	18.0	20.0	24.5	0	0	1
NEO1	Q92859	Neogenin	21.9	18.0	23.6	1	0	1
GANAB	Q14697	Neutral alpha-glucosidase AB	19.7	18.0	25.3	0	0	2
NME2	P22392	Nucleoside diphosphate kinase B	20.7	20.0	22.4	0	0	2
PAPPA	Q13219	Pappalysin-1	28.9	28.8	32.0	14	12	31
PXDN	Q92626	Peroxidasin homolog	32.6	32.4	33.7	49	48	58
PDGFD	Q9GZP0	Platelet-derived growth factor D	20.3	22.9	28.0	0	1	5
PLEC	Q15149	Plectin	19.7	27.1	30.0	1	8	28
PFN1	P07737	Profilin-1	18.0	19.8	24.8	0	0	1
PDIA3	P30101	Protein disulfide-isomerase A3	20.3	18.0	22.2	0	0	1
S100A10	P60903	Protein S100-A10	19.4	18.0	25.4	0	0	1
SEC23A	Q15436	Protein transport protein Sec23A	18.0	18.0	26.2	0	0	2
7WNT5A	P41221	Protein Wnt-5a	27.4	28.3	30.8	4	5	9
PTPRD	P23468	Receptor-type-tyrosine-protein phosphatase delta	20.9	18.0	23.2	0	0	1
SPTAN1	Q13813	Spectrin alpha chain, non-erythrocytic 1	25.1	25.9	28.6	4	4	12
SPTBN1	Q01082	Spectrin beta chain, non-erythrocytic 1	24.7	25.3	28.9	2	2	11
SPON1	Q9HCB6	Spondin-1	21.1	20.3	28.8	0	0	5
STC1	P52823	Stanniocalcin-1	27.4	26.2	29.0	2	2	3
STC2	O76061	Stanniocalcin-2	26.2	26.6	28.6	2	2	4
SRPX	P78539-5	Sushi repeat-containing protein SRPX	26.6	26.5	29.6	4	3	9
VCP	P55072	Transitional endoplasmic reticulum ATPase	19.4	20.4	29.2	0	1	8
TNFAIP6	P98066	Tumor necrosis factor-inducible gene 6 protein	28.4	28.6	30.7	8	7	11
**DIFFERENTIALLY UPREGULATED RELATIVE TO WEEK 1**								
ACTR3	P61158	Actin-related protein 3	28.16	28.5	29.6	6	6	7
ARPC1B	O15143	Actin-related protein 2/3 complex subunit 1B	25.1	25.7	26.6	2	2	3
ENO1	P06733	Alpha-enolase	27.4	27.5	28.6	3	4	6
ANXA5	P08758	Annexin A5	24.5	26.4	28.8	1	5	7
AP2B1	P63010	AP-2 complex subunit beta	21.1	22.9	25.0	1	1	2
28CLTC	Q00610	Clathrin heavy chain 1	22.8	26.0	28.1	1	4	7
C1S	P09871	Complement C1s subcomponent	31.2	31.8	32.6	19	20	21
CXCL5	P42830	C-X-C motif chemokine 5	23.3	25.8	28.1	1	2	3
ART4	Q93070	Ecto-ADP-ribosyltransferase 4	18.0	25.7	25.3	0	1	1
6F7TL	P02792	Ferritin light chain	18.0	25.7	26.9	0	1	2
FN1	P02751	Fibronectin	38.9	39.5	40.0	138	149	160
LGALS3BP	Q08380	Galectin-3-binding protein	25.1	26.2	28.1	2	3	5
PYGB	P11216	Glycogen phosphorylase	24.1	25.4	26.9	1	2	2
GVINP1	Q7Z2Y8	Interferon-induced very large GTPase 1	22.6	25.2	26.3	1	1	1
LAMA2	P24043	Laminin subunit alpha-2	32.0	33.0	33.9	59	73	89
LAMC1	P11047	Laminin subunit gamma-1	32.6	33.3	34.2	46	52	58
S100A6	P06703	Protein S100-A6	24.9	26.6	27.9	1	1	2
CXCL12	P48061	Stromal cell-derived factor 1	18.0	21.5	25.7	0	1	1
TFPI	P10646	Tissue factor pathway inhibitor	22.5	20.0	22.7	1	0	1
TPI1	P60174	Triosephosphate isomerase	18.0	18.0	24.6	0	0	1
UCN3	Q969E3	Urocortin-3	18.0	18.0	24.9	0	0	1
VIM	P08670	Vimentin	29.1	30.2	32.0	9	14	20
**DIFFERENTIALLY UPREGULATED RELATIVE TO WEEK 2**								
ANGPT1	Q15389	Angiopoietin-1	28.3	28.2	29.5	6	7	8
AP2A1	O95782	AP-2 complex subunit alpha-1	22.3	18.0	25.6	1	0	2
APOD	P05090	Apolipoprotein D	30.2	25.6	31.0	6	4	6
HSPG2	P98160	Basement membrane-specific heparan sulfate proteoglycan core protein	32.2	30.7	32.7	53	28	57
COL12A1	Q99715	Collagen alpha-1(XII) chain	32.4	30.9	33.6	74	45	87
COL16A1	Q07092	Collagen alpha-1(XVI) chain	25.5	19.7	25.1	2	0	2
EEF1A1	P68104	Elongation factor 1-alpha 1	27.5	27.5	29.1	4	3	6
EEF2	P13639	Elongation factor 2	27.7	27.3	28.5	8	6	9
EMILIN1	Q9Y6C2	EMILIN-1	28.7	29.3	30.8	11	13	18
CXCL1	P09341	Growth-regulated alpha protein	26.5	26.4	28.3	2	1	3
PYGB	P11216	Glycogen phosphorylase	25.4	24.1	26.9	2	2	2
SPON1	Q9HCB6	Spondin-1	21.1	20.3	28.8	22	12	23
FBN1	P35555	Fibrillin-1	33.3	31.7	33.4	48	32	52
FBN2	P35556	Fibrillin-2	33.2	31.1	32.6	53	32	46
GAS6	Q14393-3	Growth arrest-specific protein 6	29.9	29.4	26.9	11	10	4
HOXC10	Q9NYD6	Homeobox protein Hox-C10	30.5	31.6	33.9	1	1	1
MYH9	P35579	Myosin-9	28.1	26.5	29.0	7	3	9
GANAB	Q14697	Neutral alpha-glucosidase AB	19.7	18.0	25.3	11	13	18
NID2	Q14112	Nidogen-2	31.0	29.1	32.0	22	12	24
PGM1	P36871	Phosphoglucomutase-1	26.6	25.0	26.7	3	2	4
PCOLCE2	Q9UKZ9	Procollagen C-endopeptidase enhancer 2	27.0	25.0	26.8	3	2	3
PKM	P14618	Pyruvate kinase PKM	30.5	29.5	30.8	11	10	14
QSOX1	O00391	Sulfhydryl oxidase 1	27.8	27.1	29.0	5	6	8
SOD2	P04179	Superoxide dismutase	27.3	28.0	30.8	1	1	2
SNED1	Q8TER0	Sushi, nidogen and EGF-like domain-containing protein 1	26.0	25.6	27.8	4	3	7
THBS1	P07996	Thrombospondin-1	36.4	35.3	37.2	20	15	21
TGFBI	Q15582	Transforming growth factor beta-induced protein ig-h3	32.1	31.1	32.8	2	1	16
TUBB4B	P68371	Tubulin beta-4b chain	22.1	19.8	23.6	2	1	3
WISP2	O76076	WNT1-inducible-signaling pathway protein 2	23.1	26.1	29.1	2	1	4

*The table presents proteins significantly upregulated in sEVs isolated at week 3 when compared to weeks 1 and 2*.

a * represents the average number of unique peptides identified in three biological repeats*.

b * represents the average log(2) transformed signal intensity*.

*For a full list of proteins identified in the study please see Supplementary Table 1*.

*Full accompanying statistical data can be found in Supplementary Table 2*.

**Figure 5:**
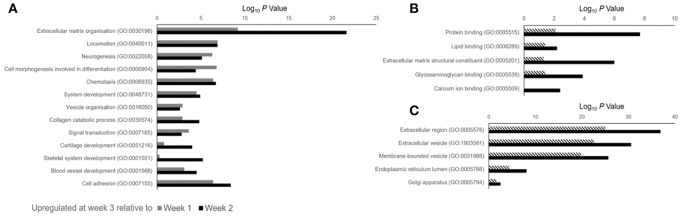
Gene ontology (GO) analysis of proteins identified as being differentially upregulated in sEVs isolated during the third week of osteoblast differentiation relative to those isolated at weeks 1 (dashed line) and 2 (block color). A total of 116 proteins were identified, with 87 and 99 proteins found to be differentially upregulated when compared with sEVs isolated at weeks 1 and 2, respectively. **(A)** Biological processes, **(B)** molecular mechanisms and **(C)** cellular component predicted for proteins identified at week 3.

Data outlined in Figures 3, 4 demonstrated much of the significant changes in sEV protein content occurred at week 3, when mineralization was most advanced. Few changes in protein intensity were observed between weeks 1 and 2 (79% Pearson correlation). As such, we carried out gene ontology (GO) analysis using the PANTHER (protein annotation through evolutionary relationship) classification system to identify differences in predicted biological function, molecular mechanism and cellular compartment of proteins upregulated at 3. Based on our stringent exclusion criteria, a total of 116 proteins were found to be upregulated or exclusive to vesicles isolated during the third week of osteogenic differentiation. Of these 116 proteins 56% were non-specifically upregulated relative to vesicles isolated at both week 1 and 2. GO analysis of biological process revealed overlapping functions in extracellular matrix organization and cell adhesion, with both processes particularly upregulated at week 3 when compared with week 2 (Figure 5A). In addition, proteins identified in sEVs at week 3 aligned with cartilage and skeletal system development. Again, these differences were particularly evident when compared with vesicles derived at week 2. Molecular mechanisms associated with these proteins correlated with the binding of lipids, calcium and glycosaminoglycans (Figure 5B). These proteins were predicted to localize within the extracellular matrix and a higher proportion of these proteins derived from the endoplasmic reticulum (Figure 5C).

## Discussion

We have previously shown that osteoblasts secrete mineralizing sEVs into the culture medium and that administration of these particles within MSC cultures is able to enhance differentiation (Davies et al., 2017). The outcomes of this study contribute further insight on how howthe morphology and biological content of these complex nanoparticles changes as mineralization advances (Xiao et al., 2007; Morhayim et al., 2015). This could have considerable implications relating to the prospective application of sEVs in regenerative medicine as well as the reproducibility of research findings between studies. In the present study we isolated sEVs from the culture media of mineralizing primary osteoblasts over a period of 21 days under chemically-defined conditions. We identified the presence of sEVs during early (week 1), mid (week 2), and late (week 3) matrix mineralization and noted a significant reduction in particle concentration at week 3. At each time point we observed a consistent bimodal size distribution indicative of a heterogeneous population of microvesicle-like (100–1000 nm), as well as exosome-like (30–150 nm) particles (Raposo and Stoorvogel, 2013). As osteoblast differentiation advanced EVs became less polydisperse, with the presence of two distinct populations becoming more apparent. When visualized using TEM, morphological differences were evident between vesicles generated during early (week 1) and terminal (week 3) stages of osteoblast differentiation, with visual indications of compromised structural integrity evident during week 3. This is likely related to the progressive formation of mineral crystals previously shown to radially bud and perforate the vesicle membrane (Eanes, 1992; Skrtic and Eanes, 1992). These growing crystals associate with the surrounding osteoid to form mineralized nodules that propagate along an external collagen template (Hasegawa et al., 2017). Such findings align with observations reported in a model of rat tibial regeneration, where evidence of vesicle rupture was reported following 21 days, in line with proximity to the calcified front (Sela et al., 1992). Deformation of these vesicles may also account for the significant reduction in particle number detected at week 3 and the distinct absence of particles over 330 nm in size, which may have ruptured (Amir et al., 1988). The absence of a visually distinctive plasma membrane is likely to be related to the commercial method of precipitation applied to isolate the vesicles (Martins et al., 2018).

In addition to morphological variations in vesicle profiles, we were able to identify a correlation between total sEV protein number and osteoblast differentiation status, as indicated by a declining Pearson correlation coefficient of 0.79 (week 1 vs. 2), 0.60 (week 2 vs. 3), and 0.46 (week 1 vs. 3). Gene ontology analysis highlighted the significant differences in protein expression at week 3 correlated with an enrichment in extracellular, vesicular and membrane-bound proteins with biological functions in extracellular matrix organization, angiogenesis, and skeletal development. These results contribute evidence to demonstrate osteoblast sEVs are dynamic units that have temporally defined roles in matrix development. To date, relatively few studies have analyzed the sEV proteome. The first study to do so compared vesicles isolated from culture medium using ultracentrifugation with matrix vesicles embedded in the extracellular matrix. In this study the 544 proteins were identified in sEVs isolated from immortalized mineralizing MC3T3-E1 cells, whereas the present study documented only 249 common proteins among sEVs isolated over a period of 3 weeks. However, a number of common extracellular matrix proteins (Emilin-1, MMP-2, Periostin, Thrombospondins, and Decorin), enzymes (Aminopeptidase-N, superoxide dismutase and Cathepsin proteins) and membrane proteins (Integrins αV and β3) are common to sEVs isolated in both studies. Further correlation exists in the presence of cytoskeletal (α-actinin-1 and−4, Vinculin and Gelsolin), signaling (GTP-binding proteins, 14-3-3 proteins and Rab family proteins) and calcium regulating (Annexin and S100 proteins) proteins (Xiao et al., 2007). The second study applied ultracentrifugation to isolate sEVs from the culture medium of mineralizing human SV-HFO fetal pre-osteoblast cells and, to our knowledge, is the only other study to have profiled sEVs over the course of mineralization (Morhayim et al., 2015). Morhayim et al. identified a total of 1116 proteins, a number of which represented osteoblast-related proteins linked to skeletal development, differentiation and calcium ion binding. These included a number (30%) of osteoblast-EV specific proteins, which were not observed in our own study. Variations observed in total protein content observed between studies are likely manifold, with differences in cell type and sEV isolation procedure and time of isolation being perhaps most significant. However, the robustness of our approach is demonstrated by the identification of common annexin (ANXA2, ANXA6), tetraspanin (CD81) and metabolic (GAPDH, LDHA) vesicle markers (Morhayim et al., 2015). Further, EVs isolated in the present study contained 60% of the most 100 most prevalent EV proteins as published by Vesiclepedia (http://microvesicles.org/extracellular_vesicle_markers). Perhaps most interestingly, in line with the findings of Morhayim et al. we provide evidence through gene ontology analysis to indicate that bone-related processes are one of several primary functions of sEVs.

In addition to the protein signature of sEVs,time dependent changes in the lipid profile have been previously demonstrated. These findings were described in a rodent model of endosteal repair, where variations in phospholipids, including phosphotidylcholine (PC), phosphotidylserine (PS) and sphingosine were found to correlate with the stage of regeneration (Schwartz et al., 1989). Since the organization of phospholipids within the EV membrane is critical for driving mineral formation via the NCC, variations in content will inevitably influence protein interactions that could impact the nucleational capacity of vesicles and their association with the extracellular matrix. However, to our knowledge, no study has yet profiled changes in the protein content of osteoblast-derived vesicles during the course of mineralization. Central to the NCC is the annexin family of calcium regulated phospholipid-binding proteins, which localize to the vesicle lumen, outer surface and inner leaflet. Members of the annexin family are proposed to associate with Ca^2+^, inorganic phosphate (Pi) and PS to configure an optimal environment for the nucleation of mineral crystals. It is hypothesized that this interaction drives the transition of amorphous calcium phosphate through a series of intermediate phases to generate thermodynamically stable hydroxyapatite crystals (Anderson, 1995). However, annexin proteins are also likely to have additional roles in the binding and channeling of calcium ions. We have previously shown that annexin proteins I, II and VI are significantly upregulated in mineralizing sEVs derived from the culture medium of mineralizing osteoblasts (Davies et al., 2017). The pro-osteogenic effects of these proteins has been further demonstrated in synthetic models of the NCC, where the incorporation of Annexin-A5 promoted a significant increase the rate and total amount of mineral formed when incubated with synthetic cartilage lymph (Genge et al., 2007). In the present study we provide evidence to indicate a temporal relationship between the profile of annexins and the osteoblast maturity, with annexin-A2, -A5 and -A6 significantly upregulated in sEVs during the third week of osteoblast differentiation, where significant mineralization was evident (Figure 1). Such variation in proteins either directly or indirectly associated with the NCC will have implications when considering the potential application of osteoblast-derived vesicles for regenerative applications, where it will be therapeutically advantageous to select for the most osteo-inductively potent particles (Bjørge et al., 2018).

Temporal differences in annexin proteins also reflect biochemical changes in the composition of the developing ECM indicative of maturation that are, at least in part, related to collagen composition. It has been proposed the collagen-interacting properties of vesicles may function to align them within the matrix and provide a template for mineralization (Bottini et al., 2018). For instance, Annexin A2 mediates the secretion of collagen type-VI (Dassah et al., 2014), which functions by bridging cells with the surrounding connective tissue and organizing the three-dimensional architecture of bone (Cescon et al., 2015). In the present study we show that type-VI collagen was significantly upregulated in sEVs during the third week of mineralization *in vitro*, thus supporting findings by Lamandé et al. (2006) demonstrating the formation of stable collagen-VI helices by a SaOS-2 osteosarcoma cell line occurs later in the differentiation pathway (Lamandé et al., 2006). Additional collagen subtypes, including type-I collagen were found to be associated with vesicles but did not vary over the course of mineralization. Annexin A2 and A6, which were also found to be upregulated at week 3, are known to interact with chondroitin sulfate, a structural component of cartilage and an important mediator in bone regeneration, in a Ca^2+^-dependent manner (Ishitsuka et al., 1998; Takagi et al., 2002). While the interaction between annexin A6 and protein kinase C α has been shown to mediate terminal differentiation and mineralization events of hypertrophic chondrocytes by channeling the influx of Ca^2+^ ions (Minashima et al., 2012). As such, temporal variations observed in annexins and their defined interactions with collagens, which are fundamental to the development of the mineralizing ECM, implicate sEVs as mediators of changes in the ECM required for the transition between hypertrophic cartilage and endochondral bone.

In addition to specific interactions with collagenous and non-collagenous components of the ECM, vesicles function in the transportation of collagens and matrix proteins from the endoplasmic reticulum into the extracellular environment. This is achieved through coupling with coat protein carriers, which include COPII carriers such as Sec23a (Malhotra and Erlmann, 2015). In the present study, the inner coat protein Sec23a was found to be upregulated in vesicles during the third week of mineralization, correlating with an enhanced presence of collagen type-VI. Furthermore, COPII vesicles utilized for the translocation of collagens into the ECM are typically under 100 nm, which correlates with the smaller subpopulation of particles identified in the present study (Weeks 1 and 2, 44 nm; Week 3, 54 nm – Figure 2D). Although we are aware that further investigation will be required to delineate the mechanisms by which sEVs deliver collagen subunits to drive development of the ECM, we believe that our initial findings are of important biological relevance and propose that osteoblasts secrete distinct vesicle subsets with discrete roles during osteogenesis. In addition to collagens, profiling vesicles over the course of a three-week differentiation period revealed the presence of additional proteins known to be localized within the endoplasmic reticulum, including reticulocalbin, calumenin, reticulocalbin-3, endoplasmin, and translational endoplasmic reticulum ATPase. These findings provide further support to the theory that at least a subset of osteoblast-derived vesicles derive from the trans-Golgi network and are trafficked to the plasma membrane via interaction with members of the Rab family of small GTPases where they fuse and are released into the extracellular environment (Stenbeck and Coxon, 2014). Furthermore, the contribution of ER-associated vesicles appears to be dependent on the differentiation status of the osteoblast, with proteins such as reticulocalbin, endoplasmin and calumenin largely down-regulated during the third week of mineralization.

Further to the capacity of Annexin A2 to mediate the secretion of collagen type-VI, this calcium-binding protein also interacts with the Wnt signaling pathway inhibitor sclerostin, which has led to the suggestion that this protein may function in the regulation of osteogenesis (Devarajan-Ketha et al., 2012). The Wnt family of secreted glycoproteins is critical for regulating bone homeostasis through β-catenin-dependent canonical and β-catenin independent non-canonical pathways. In the present study we identified the presence of Wnt5a and the Wnt-1-inducible signaling pathway protein (WISP-2) in sEVs and documented significant upregulation during the third week of mineralization (Table 1), when a calcified ECM was evident (Figure 1). WISP-2 has previously been shown to promote cell adhesion and differentiation depending on whether it is secreted or localized within the cytoplasm (Grünberg et al., 2014). However, the precise function of this protein in bone tissue is far less well-understood than its sister protein, WISP-1, which has well-documented roles in osteogenic differentiation and bone repair (Maiese, 2016). Wnt5a functions in the regulation of non-canonical Wnt signaling with a well-established role in coordinating osteoclastogenesis through receptor tyrosine kinase-like orphan receptor 2 (Ror2)/ c-Jun N-terminal kinase signaling (Hasegawa et al., 2017). The Wnt5a ligand has also been implicated in the onset of pathological vascular calcification via its interaction with low density lipoprotein receptor-related protein-1 (Woldt et al., 2012). Homozygous deletion of Wnt5a has been shown to reduce the expression of critical pro-osteogenic genes such as ALP, osterix and osteocalcin, as well as the formation of mineralized nodules (Okamoto et al., 2014). Previous research has identified vesicles as vehicles for the systemic delivery of hydrophobic Wnt proteins in a diverse range of cell types including human umbilical cord MSCs (Zhang et al., 2015), cortical neurons (Tassew et al., 2017) and macrophages (Menck et al., 2013). However, to our knowledge, no previous studies have acknowledged the presence of these critical homeostatic proteins in sEVs. As such, we present initial findings to suggest that sEVs may function in the activation of non-canonical Wnt signaling required for osteoclastogenesis and the maintenance of bone homeostasis. However, additional studies will be required to confirm this hypothesis.

## Conclusions

Findings from the present study demonstrate that the secretion of osteoblast vesicles is a dynamic and temporal process, which is dependent on the mineralization status of the ECM. sEVs contained many proteins implicated in the nucleation and propagation of mineral in developing hard tissues and shared common morphological and biological features reported for matrix-bound vesicles. The localization of osteogenic signaling proteins and postulated components of the NCC increased as mineralization advanced. Of significance was the upregulation of annexin family members and Wnt signaling proteins in sEVs generated during week 3, since these proteins have defined roles in osteogenesis. Our findings shed light on fundamental mechanisms governing vesicle-mediated mineralization, identifying it as a dynamic process whereby progressive variations in sEV morphology and protein content mirror developmental changes matrix organization. Findings also have implications when considering the selection and application of osteoblast-derived sEVs in regenerative medicine and further studies will be required to determine the pro-osteogenic properties of temporally isolated sEV fractions when administered *in vivo*.

## Ethics Statement

This study was approved by the University of Birmingham UK Research Ethics Committee (Reference: 16/SS/0172). Written informed consent was obtained from all participants.

## Author Contributions

OD conception of study. OD, SC, SJ, ED, and LG experimental design and manuscript preparation. OD, IA, AM, SJ, ED, and MC experimental work and data analysis. LH computational data analysis.

### Conflict of Interest Statement

The authors declare that the research was conducted in the absence of any commercial or financial relationships that could be construed as a potential conflict of interest.

## Supplementary Material

Supplementary Image 1 Additional TEM images of sEV morphology.

Supplementary Table 1 A full list of proteins identified in the present study.

Supplementary Table 2 Statistical information relating to differentially upregulated proteins.
