# Corrigendum: Osteoblast-Derived Vesicle Protein Content Is Temporally Regulated During Osteogenesis: Implications for Regenerative Therapies

**DOI:** 10.3389/fbioe.2019.00392

**Published:** 2019-12-06

**Authors:** Owen G. Davies, Sophie C. Cox, Ioannis Azoidis, Adam J. A. McGuinness, Megan Cooke, Liam M. Heaney, Edward T. Davis, Simon W. Jones, Liam M. Grover

**Affiliations:** ^1^School of Sport, Exercise and Health Sciences, Loughborough University, Loughborough, United Kingdom; ^2^School of Chemical Engineering, University of Birmingham, Birmingham, United Kingdom; ^3^Physical Sciences for Health Doctoral Training Centre, University of Birmingham, Birmingham, United Kingdom; ^4^Royal Orthopaedic Hospital, Birmingham, United Kingdom; ^5^Institute of Inflammation and Ageing, University of Birmingham, Birmingham, United Kingdom

**Keywords:** vesicle, mineralization, annexin, collagen, osteoblast, nano

“Edward T. Davis” and “Simon W. Jones” were not included as authors in the published article. The author list has been updated and the corrected **Author Contributions** statement appears below.

“OD conception of study. OD, SC, SJ, ED, and LG experimental design and manuscript preparation. OD, IA, AM, SJ, ED, and MC experimental work and data analysis. LH computational data analysis.”

Additionally, in the original article, there was a mistake in Table 1 as published. Incorrect primers were included. The corrected Table 1 appears below.

**Table 1:** Primer sequences and accompanying gene accession numbers.

**Gene**	**Forward**	**Reverse**	**Accession No**.
ALP	CTTGGGCAGGCAGAGAGTA	AGTGGGAGGGTCAGGAGAT	NM_000478
BGLAP	GGCACCCTTCTTTCCTCTTC	TTCTGGAGTTTATTTGGGAGCA	NM_199173
BSP	GAGGTGATAGTGTGGTTTATGGA	TGATGTCCTCGTCTGTAGCA	NM_00104005
COL1A1	AGACAGTGATTGAATACAAAACCA	GGAGTTTACAGGAAGCAGACA	NM_000088

The authors apologize for these errors and state that it does not change the scientific conclusions of the article in any way. The original article has been updated.

