# Supplementary figures and images for: Osteoblast-Derived Vesicle Protein Content Is Temporally Regulated During Osteogenesis: Implications for Regenerative Therapies
Source: Front Bioeng Biotechnol. 2019 May 1;7:92. doi: 10.3389/fbioe.2019.00092 (PMC6504811; doi:10.3389/fbioe.2019.00092)

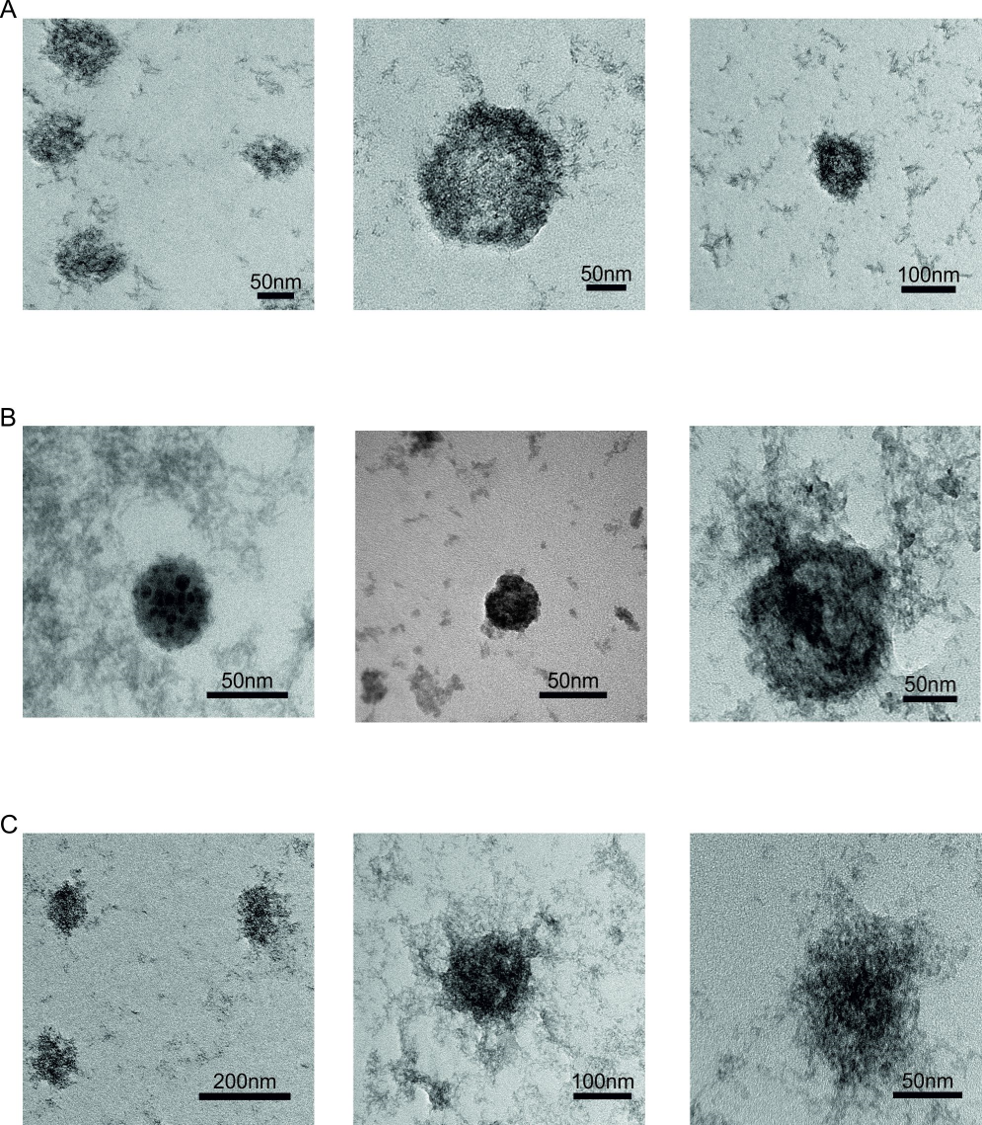

Supplement: Supplementary Image 1 — Additional TEM images of sEV morphology. [file Image_1.TIF]
